# Tailoring the taste of cultured meat

**DOI:** 10.7554/eLife.98918

**Published:** 2024-05-30

**Authors:** Gyuhyung Jin, Xiaoping Bao

**Affiliations:** 1 Davidson School of Chemical Engineering, Purdue University West Lafayette United States

**Keywords:** muscle cells, intramuscular fat, transdifferentiation, meat, Chicken

## Abstract

A new protocol can customize the flavor of lab-grown meat by controlling the level of fat deposited between muscle cells.

**Related research article** Ma T, Ren R, Lv J, Yang R, Zheng X, Hu Y, Zhu G, Wang H. 2024. Transdifferentiation of fibroblasts into muscle cells to constitute cultured meat with tunable intramuscular fat deposition. *eLife* **13**:RP93220. doi: 10.7554/eLife.93220.

Although the idea of growing meat in a laboratory may seem like science fiction, over a decade has passed since the first synthetic beef burger was unveiled in 2013 (Kupferschmidt, 2013). Moreover, in June 2023, two companies – Upside Foods and GOOD Meat – were granted approval by the Food and Drug Administration (FDA) to sell ‘cultured meat’ products in the United States (Wiener-Bronner, 2023).

While producing meat in a laboratory could eventually lead to a reduction in livestock farming, which would bring benefits in terms of improved animal welfare and reduced environmental impact (Post, 2012), the process is not free from challenges. For instance, there are concerns around food safety, the high cost of production, and whether the public will accept meat that has been artificially made. Also, if the cultured meat does not taste exactly like the real deal, consumers may turn away. Moreover, cultured meat products will have to compete against other, more affordable options, such as plant-based burgers.

A compelling advantage of cultured meat is that its texture, flavor and nutritional content can be tailored during production (Broucke et al., 2023). Lab-grown meat is made by isolating cells from the tissue of a live animal or fertilized egg, and differentiating them into muscle, fat and connective tissue. By altering the composition of cells generated during this process, researchers could make lab-grown meat that is personalized to an individual’s taste. However, combining and co-culturing the different source cells needed to produce these three components is no easy task. Now, in eLife, Hen Wang and co-workers – including Tongtong Ma, Ruimin Ren and Jianqi Lv as joint first authors – report an innovative approach for customizing cultured meat using just fibroblasts from chickens (Ma et al., 2024; Figure 1).

**Figure 1.**
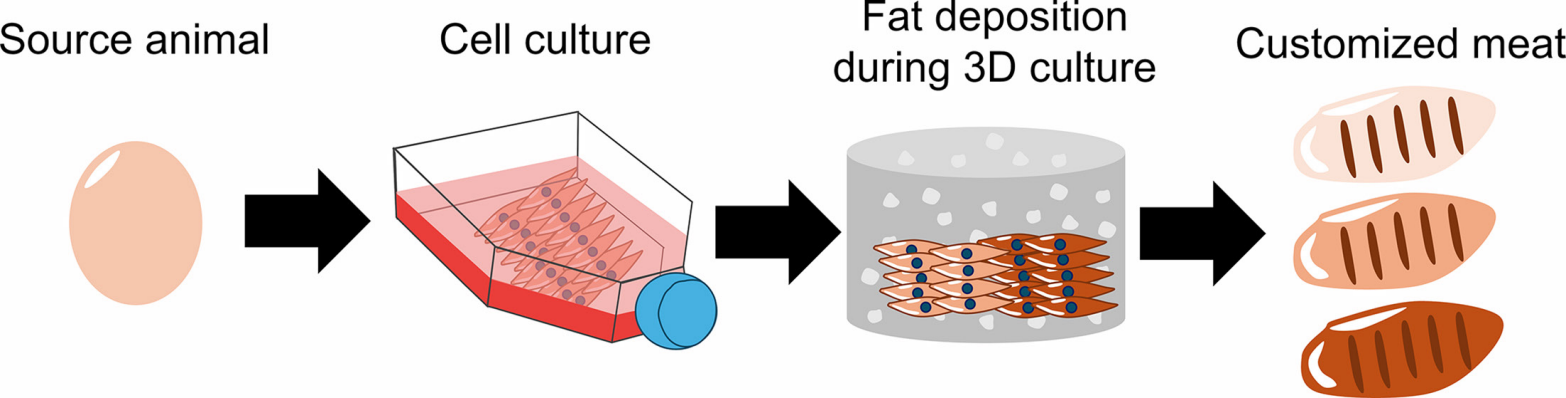
How cultured meat can be customized to enhance flavor. First, fibroblasts were sourced from the fertilized eggs of chickens and grown on a flat surface. The cells were then implanted into a three-dimensional scaffold made up of hydrogel (grey cylinder with white dots), where they were differentiated into muscle cells. The differentiated fibroblasts were then supplemented with insulin and fatty acids to induce the deposition of fat between the muscle cells. The fat content of meat influences how it tastes and smells: by changing the amount of insulin and fatty acids, Ma et al. were able to produce cultured meat with low (beige), medium (light brown), or high (dark brown) levels of fat content.

Image Credit: Gyuhyung Jin (CC BY 4.0).

First, the team (who are based at Shandong Agricultural University and Huazhong Agricultural University) optimized the culture conditions for the chicken fibroblasts. This included changing the composition of the medium the cells were fed, and developing a three-dimensional environment, made of a substance called hydrogel, that the fibroblasts could grow and differentiate in. Ma et al. then used a previously established protocol to activate the gene for a protein called MyoD, which induces fibroblasts to transform into muscle cells (Ren et al., 2022). Importantly, the resulting muscle cells exhibited characteristics of healthy muscle cells and were distinct from muscle cells that appear during injury.

While muscle is a fundamental component of meat, the inclusion of fat and extracellular matrix proteins is essential for enhancing flavor and texture (Fraeye et al., 2020). To stimulate the formation of fat deposits between the muscle cells, the differentiated chicken muscle cells were exposed to fatty acids and insulin. This caused the cells to produce lipid droplets, signifying that the process of fat synthesis had been initiated. Measuring the level of the lipid triglyceride revealed that the fat content of the cultured fibroblasts was comparable to that in real chicken meat, and even climbed two to three times higher when insulin levels were increased.

Ma et al. then validated that the chicken fibroblasts were also able to produce various extracellular matrix proteins found in connective tissue. This is critical for forming edible cultured meat products as the tissue surrounding muscle cells gives meat its texture and structural integrity.

The study by Ma et al. underscores the potential for modulating key components (such as muscle, fat, and extracellular matrix proteins) to produce high quality lab-grown meat that is palatable to consumers. It also demonstrates how the important components of meat can be produced from just a single source of fibroblasts, rather than mixing multiple cell types together. As this is a proof-of-concept study, many of the issues associated with producing cultured meat for a mass market still persist. These include the high cost of components such as insulin, and concerns around the use of genetically modified cells: moreover, potentially toxic chemicals are required to activate the gene for MyoD.

Nevertheless, advancements in biotechnology, coupled with the recent FDA approval for genetically modified cells in lab-grown meat production (Martins et al., 2024), signal a promising future for the cultured-meat market. In the future, genetic, chemical and physical interventions may be used to precisely design other bioactive compounds found in meat, in addition to fat and muscle (Jairath et al., 2024). It may not be long until personalized cultured meat products with finely-tuned flavors, textures, and nutrients are available for sale.
